# End-of-life care at home: Dignity of family caregivers

**DOI:** 10.1177/09697330241241773

**Published:** 2024-04-08

**Authors:** Katrine Staats, Kristin Jeppestøl, Bente Egge Søvde, Bodil Aarmo Brenne, Anett Skorpen Tarberg

**Affiliations:** 158935 OsloMet – Oslo Metropolitan University; 243285 University of Agder; 150769 Western Norway University of Applied Sciences; 1786 Nord University; 8018 NTNU- Norwegian University of Science and Technology

**Keywords:** dignity in care, end-of-life issues, ethics of care, family caregivers, home care, qualitative research

## Abstract

**Background:**

Healthcare services are increasingly being shifted to home settings for patients nearing end-of-life. Consequently, the burden on family caregivers is significant. Their vulnerable situation remains poorly understood and there is little information available regarding their experiences of dignity.

**Aim:**

This study seeks to understand the experiences of family caregivers related to dignity and loss of dignity, aiming to provide a deeper insight into their situation when caring for a home-dwelling family member nearing end-of-life.

**Research design and participants:**

This exploratory study consists of a second analysis combining data from two primary studies, including 24 family caregivers of a family member nearing end-of-life, and is founded upon Gadamer’s philosophical hermeneutics.

**Ethical considerations:**

Approval was obtained from the Norwegian Agency for Shared Services in Education and Research and was based on voluntary participation, informed consent, and confidentiality.

**Results:**

The following three main themes were identified: Having a meaningful existence within the living environment, being seen and valued as a family caregiver in relation with others and suffering in a space of loneliness. These contextual, relational, and existential perspectives were found to be closely interrelated.

**Conclusion and final considerations:**

The dignity of family caregivers was closely tied to being seen as unique individuals, not merely caregivers, thereby requiring healthcare professionals (HCPs) to understand their personal needs. This study highlights the emotional distress and loneliness family caregivers feel in their dual role within the healthcare system, thereby calling for HCPs to adopt an attitude of gentleness and recognition to impart dignity-preserving care in homecare practices.

## Introduction

Internationally, healthcare services are gradually being transferred to the patients’ homes because of an ongoing demographic shift.^1,2^ The opportunities for professional end-of-life care at home varies substantially; however, most people wish to spend their last days of life in familiar surroundings. This indicates that patients are increasingly coping with their illnesses at their own homes, which requires a tremendous effort from their family members.^3^ Changing from the role of family member into the role of the *family caregiver*, they often feel unprepared to provide care for their loved ones, as it exceeds their resources, creates insurmountable practical and emotional challenges, and significantly impacts their lives.^4,5^ Moreover, the family caregivers may feel incapable and lonely in making ethical decisions regarding the illness itself and imminent death and often lack of crucial information to master such relevant care.^6,7^ Consequently, the caregiving feels overwhelming, and family caregivers may undergo negative experiences with regard to the caring situation.^8^ This responsibility represents a significant and unrecognized burden that may stretch beyond breaking point for them.^9^

## Background

In this study, we use the term family caregivers to include an entire breadth of relationships that are closely related to patients’ lives. A family caregiver is designated by the patient and is often defined as being the one providing a broad range of assistance for a patient in need of care.^10^ In the Norwegian context and according to its legal definition,^11^ the family caregiver is the person to be informed about the patient’s health condition only when the patient permits the sharing of such information. This implies that the family caregiver plays an important role in patient care; it also reveals how they might experience lack of individual rights.^12^ Furthermore, family caregivers express how difficult it is to live independently in a mutual fragile life situation, as they feel their lives are intertwined with that of the patient.^13^ This reflects the importance of supporting the family caregivers in their demanding and invisible role in which they experience a high level of psychological distress.^14,15^ This is also related to how the care for family caregivers is almost absent within such settings.^5^ Despite this portrayal of the family caregiver’s situation of constant worrying, studies reveal how providing care for a close family member nearing end-of-life at home also can involve positive experiences.^8,16,17^ Woodman et al.^18^ described how families experienced a sense of ‘togetherness’ when they cared for an ill family member in their home and allowed them to demonstrate their love for them. This harmonizes with the overall goal of palliative care – that is, to improve the quality of life, not only for the patient but also for their families.^19^

In end-of-life care, the concept of dignity is crucial and greatly impacts the quality of care provided by family caregivers and healthcare professionals (HCPs).^20,21^ Dignity also plays a key role in the mutual relationship between the patient and the family caregiver – indicating the importance of relieving the caregiver’s distress to promote dignity for both the patient nearing end-of-life and their caregiver.^22,23^ Prado et al.^24^ emphasize that when HCPs provide the right support to family caregivers who experience the process of end-of-life care at home, the likelihood of a dignified death for the patient increases. However, as stated by the World Health Organization (WHO),^25^ not only the patient but all people, in this case family caregivers, have the right to be treated with dignity. According to Care theorist Katie Eriksson,^26^ this implies that all human beings are unique and have inherent dignity. To illustrate this form of dignity, Pols et al.^27^ reveal how a family caregiver caring for a close family member mirrors experiences of dignity with their own values in life. For example, to ignore the threatened dignity of their dying relative would also imply a lack of dignity of themselves. Despite most research on dignity and family caregiver appearing to give attention largely to patients’ dignity, a few studies have explored how the dignity of patients was strongly interrelated to the dignity of their family caregiver.^20,28–30^ For example, when ensuring good care for the patient, despite having to give up part of their lives, it provides dignity for both the patient and the family caregiver. However, it is also found that the level of dignity-related distress is interrelated between the patient and the family caregiver. To the best of our knowledge, little information is available regarding aspects that influence dignity and loss of dignity solely of family caregivers living close to a family member nearing end-of-life within a home context. Moreover, despite an abundance of research over the last 40 years that indicates the demanding role of family caregivers, identifying it as a complex matter, their vulnerable situation remains poorly understood.^13,31^ Therefore, the aim of this study is to shed light on family caregivers in a vulnerable stage of life when caring for a family member nearing end-of-life at home, and to obtain a deeper understanding of their experiences related to dignity. The research question is as follows: How does caring for a family member nearing end-of-life at home impact family caregivers’ experiences of dignity and the loss of dignity?

## Methodology

This study has a qualitative, explorative, and descriptive design, consisting of data derived from two earlier completed studies. These studies were independently performed by the first and last author in 2017 and 2019, respectively – hereby called the primary studies. A secondary analysis is conducted, including a total of 24 family caregivers as participants (see Table 1). The aim of a secondary analysis is to address new research questions based on previous collected data.^32,33^ It is recommended to emphasize analytical techniques that are similar or close to those of the primary research, therefore, Gadamer’s hermeneutical methodology was used to interpret the transcribed text by emphasizing the hermeneutic circle.^34^ As such, we developed a deeper understanding of the meaning of the text, while continually revising and adjusting our pre-understanding.

**Table 1.** Characteristics of study participants.

	Dataset 1 (*N* = 11)	Dataset 2 (*N* = 13)
Female	9	6
Male	2	7
<40 years	2	1
41–50 years	1	3
51–60 years	3	1
61–70 years	2	4
71–80 years	3	4
Spouse	9	6
Sister	0	2
Daughter/son	2	5
Bereaved caregiver	11	0
Current caregiver	0	13

### Participants and data collection of the primary studies

Within both primary studies, we interviewed family caregivers from the patient and the relative perspective; however, in this current study, our aim is to purely focus on the family caregivers’ experiences. To understand the context and methodology in which the data were initially collected in, we will here provide a brief overview of the two primary studies:

#### Dataset 1: Silent voices: family caregivers’ narratives of involvement in palliative care^13^

This study had a qualitative design with a narrative approach^35^ aiming to explore how family caregivers experience involvement in palliative care. The research question sounded as follows: How do family caregivers experience information and involvement in the different phases of palliative care? Eleven bereaved family caregivers who lost their relative to cancer 3–12 months prior to data collection were recruited by purposive sampling between November 2016 and May 2017. Other inclusion criteria were having followed a patient closely in a palliative care trajectory, including services from both primary and specialist health care, being older than 18 years of age and being able to speak and read Norwegian. Oncology nurses assisted with the recruitment procedure and the interview took place in their homes, except from two who preferred a community institution. Individual narrative interviews were conducted and transcribed by the first author, which lasted between 50 and 180 min. Examples of questions leading the interview were (Table 2).

**Table 2.** Examples from interview Study 1.

Individual narrative interviews	- Can you tell me how you experienced the palliative care pathway?
	- How do you experience being involved in the different phases of the palliative care pathway
	- How did you experience the information you received in different phases of the palliative care pathway?

#### Dataset 2: Dignity of older home-dwelling women nearing end-of-life: Informal caregivers’ perception^29^

This study was founded upon Gadamer’s philosophical hermeneutics^34^ and aimed to explore informal caregivers’ perceptions of sources related to dignity and dignity loss in end-of-life of older home-dwelling women with incurable cancer. Thirteen family caregivers were recruited by purposive sampling between November 2018 and December 2019. Cancer coordinators assisted with the recruitment procedure, and the interview was carried out in their homes, except from two who preferred a work location. They were included if they were older than 18 years of age, had responsibility for informal end-of-life care of a woman >65 years who lived with incurable cancer at home, and were able to speak and read Norwegian. Individual in-depth interviews were conducted by the first author. All interviews were recorded, transcribed verbatim, and lasted between 50 and 81 min. Individual in-depth interviews were utilized, in which a semi-structured modifiable interview guide was focused upon (Table 3).

**Table 3.** Examples from interview Study 2.

Individual in-depth interviews	- Can you describe how it’s like to be a family caregiver when caring for your wife/mother/sister – and whether this affects your experience of dignity in your everyday life?
	- Can you describe a dignity degrading situation?
	- How do you think the municipal health service can contribute to a dignified death for your wife/mother/sister?

### Second analysis using the hermeneutical circle

In applying Gadamer’s hermeneutical methodology, the interpretation process operates on multiple levels and is an ongoing, cyclical process known as the hermeneutical circle.^34,36^ This process involves a constant dialogue between the whole and its parts, with each informing and enhancing the understanding of the other.^37^ In analysing the data for this article, the first and last authors initiated the interpretive circular process by individually reading clean, uncoded transcribed data from both primary studies. Then we moved to a detailed examination of individual parts of the text, which is the first level of interpretation. Prior to involving the remaining research team, we shared our respective reflections and questioned each other’s understanding and interpretation of the data. We also sought to find overlapping and contradictory interpretations across the two studies. Then, we turned back to the whole, now with a deeper understanding of its parts, and presented preliminary emerging patterns of meaning to the rest of the research team – within the second level of interpretation. Thereafter, we wandered further as a team from preliminary ideas and aspects of the transcribed text to the initial interpretation of the text as a whole, and then back again, which is in line with Gadamer’s hermeneutical methodology.^34^ In addition, we paid close attention to our pre-understanding when we explored and formulated themes and sub-themes as our final interpretive understanding.

#### Pre-understanding

As a research team, our pre-understanding was not neutral and distanced from the subject under investigation. All authors are registered nurses with different working experiences, and research experiences with end-of-life care related to family caregivers. Therefore, our pre-understanding was discussed and challenged to ensure transparency and trustworthiness.^38^ First, to establish *credibility*, we maintained a continuous focus on our research questions as a research team, reflecting on our diverse backgrounds and expectations to avoid rushing to conclusions during data interpretation. Second, to establish *dependability* within our study, we revised the preliminary findings several times as it challenged our initial understandings, prompting us to explore different interpretative possibilities. Third, to enhance the *confirmability* of our data, we were preocupied with allowing ourselves to detach from old preconceptions and ensure that our findings were not rooted in personal biases. Fourth, we methodically and precisely illustrated the link between the findings and the gathered data as well as provided a comprehensive account of the context in which our findings were generated, to provide *transferability*. Lastly, to enhance *authenticity*, we aimed to present the experiences and perceptions of the participants in a manner that was truthful and respectful – quoting their words verbatim. In sum, given that our pre-understanding has the potential to influence the interpretation of empirical data, we actively challenged these preconceptions by engaging in critical reflection during our research meetings which collectively contributed to the trustworthiness of our study.

#### Ethical considerations

Regarding the primary studies, all participants were considered vulnerable as they were either a current family caregiver^29^ or a bereaved family caregiver^13^ of a close family member. Therefore, ethical considerations and sensitivity were increased to protect the family caregivers during the recruitment and data collection phase. For example, they were given the time to decide whether they wanted to participate as well as given sufficient time to respond during the interviews. Moreover, first and last authors utilized their previous experiences as oncology nurses, displaying sensitivity and considering the struggle of the family caregivers. In addition, the family caregivers were offered follow-up contact by the municipality cancer nurse after the interviews. Further, all participants were provided with an information letter and an inform consent form to complete and were assured of anonymity and confidentiality. The information letters provided in both primary studies also stated that the data might be used for subsequent publications. Both studies were conducted in accordance with the Declaration of Helsinki^39^ and were approved by the Norwegian Agency for Shared Services in Education and Research (ref nr. 2016/960-25 and 138698).

## Results

**Figure 1.**
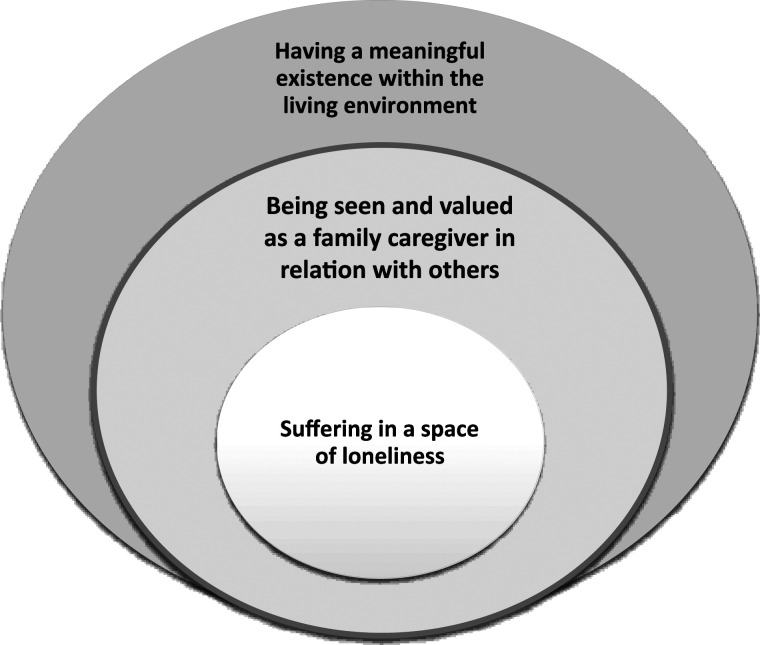
Three circles representing distinct dimensions of dignity for family caregivers.

We identified three main themes that encompassed how family caregivers experienced dignity-preserving sources and dignity loss when living close to a family member nearing end-of-life at home (Figure 1):

The circles in this figure serve to illustrate the different dimensions of dignity as experienced by the family caregivers, with each circle embodying a distinct perspective. First, the outer circle reveals how the theme *Having a meaningful existence within the living environment* visualizes contextual and fundamental sources of dignity for the family caregiver. The middle circle in the theme represents the aspect of *Being seen and valued as a* family caregiver *in relation with others,* viewed from relational and psychosocial perspectives. Finally, centre of the circle represents how the aspect of *Suffering in a space of loneliness* visualizes existential perspectives in the lives of family caregivers, which leads to loss of dignity. Movements between these circles are dynamic and illustrate how family caregivers’ experiences and perceptions can shift across these different perspectives. At times, the family caregiver might find their sense of dignity influenced by their physical living environment, as indicated by movement towards the outer circle. At other times, their interpersonal relationships or their internal existential state might play a significant role, and therefore the movement towards the middle will occur. This dynamic movement between the circles, therefore, underlines the complex and interwoven nature of dignity, and is crucial for caregivers in understanding and addressing the holistic needs of family caregivers to uphold their dignity.

### Having a meaningful existence within the living environment

The family caregivers who live close to a family member nearing end-of-life in their home experienced a meaningful existence in terms of both their role as a caregiver and in their living environment. Many of the family caregivers felt responsible for the care given to their loved ones and shared experiences regarding how they brought satisfaction in their lives. This was particularly the case for spouses who had shared many years with their sick partner, in which some described their living as a state of mutual interdependence. Despite the burden of caregiving, they experienced it as a matter of course and a means to display their love and gratitude:

For me, it is meaningful to do things for her. Then, she gets spared from what is burdensome, and she will not get so tired. It feels good. As a caregiver. Just being there. Still being a part of her life (daughter of patient, age 45).

In addition to the meaningfulness of being close to their ill family members, the experience of being independent in their home was also described as fundamental for the family caregivers. They felt like they were in charge and inviting family and friends into their own environment was easy. This was described as meaningful, but it was also related to certain expectations:

When joining my wife at the hospital, I feel like being in the background (….) But when we are in our home, it is important for me to be informed about what is going on (….) adapting to the situation here (….) Because this is our home. It is my home. And it is her home. It must be adjusted and optimized, both for me and for her. It is all about living in a valuable environment (….) (husband of patient, age 67).

A few family caregivers described how they experienced a meaningful existence only when this was a shared experience with their ill family member. When the patient experienced the environment as safe and meaningful, it was easier for the family caregiver to adapt to this feeling. However, several family caregivers described it as an at-risk situation, gradually becoming unmanageable. This implied that the family caregivers felt pressured into being in a role of caring that they lacked the strength and knowledge to fulfil. They were struggling to keep up the normality in their everyday lives and creating meaningfulness for the patient, albeit being exhausted and feeling vulnerable.

I don’t think they have thought so much about me. They see that I am up and around, and (….) I don’t think they take notice of my situation. I don’t know how it works, but they come in here, focusing on my wife. (…) I just feel I must be the strong one. I cannot break down, even though I want to do it sometimes (husband of patient, age 74).

### Being seen and valued as a family caregiver in relation with others

For the family caregivers to experience strength in their everyday life when caring for an ill patient in their home, relational aspects appeared to be crucial. This was related to both the relationship with the patient as well as with HCPs. Even though most of the family caregivers expressed a desire to be seen and valued as caregivers, the importance of being solely a family member was also present. However, this situation was indicated as being difficult to balance in their everyday lives. A few family caregivers reported certain preconditions for the role of a close caregiver, being included and informed within practical tasks and being emotionally supported. Nevertheless, most family caregivers were not aware of their position as caregivers, and, therefore, genuinely appreciated being seen as one:

All these hours when the HCPs are not here. (….) then I am here for her. I was planning to deal with this until a nurse told me, ‘You are not the nurse; you are her husband, and that is who you are supposed to be’. (…) I must say this approach felt good, it opened my eyes and got me more conscious about my role as caregiver (husband of patient, age 74).

With regard to the relationship with the patient, it was changed and imbalanced due to new roles and major changes in their lives. This could bring about feelings of guilt and powerlessness, creating difficult situations. A few family caregivers described it as entering a state of dual feelings. On the one hand, they experienced being caring and empathic and on the other hand, they felt impatient and frustrated. Furthermore, many of the family caregivers described how situations of a mutual wish to protect each other forced the family caregiver to be on their toes all the time, thereby creating further tensions in their relationship. A daughter explained this situation in the following manner:

She doesn’t want to be a burden to me (…) when she is in the hospital, she asks me not to come. Even though I have the day off and really want to be there with her (….) Then I get irritated, you know? Why can’t I come? I think it is nice to be there with her. I know she wants to spare me, but on the other hand, I also know she enjoys the time we have together (daughter of patient, age 42).

Next to the strong relationship with the patient, many of the family caregivers described the care from and connectedness with HCPs as particularly vital. As most of the family caregivers did not have any prior knowledge in nursing and care, all practical help and suggestions to make the everyday-life easier was highly appreciated. For example, when HCPs saw their needs and introduced practical aids, such as a sheet to make the movements in bed easier, the family caregivers expressed gratitude for the dedicated HCPs. With regard to the emotional support, particularly when being exhausted, being seen and valued as a caregiver was critical:

Our doctor asked me to come alone to him. He asked me directly if I was sleeping properly and told me that I had to take care of myself. I kind of saw that he asked me how I was doing by that look in his eyes. He had known me for several years (wife of patient, age 73).

While a minority of the family caregivers highlighted that such familiarity with HCPs increased the likelihood of being seen and valued as a family caregiver, most caregivers found themselves feeling lonely while caring for an ill family member at home.

### Suffering in a space of loneliness

Most of the family caregivers indicated that they felt lonely and found it difficult to express this to HCPs and other family members. Being in an unpredictable and lonely situation, many of the family caregivers reported how they were barely ever offered support or a conversation by the HCPs. Consequently, many put their worries on hold and kept up a facade of being strong in the caring situations. The comment below illustrates that they felt particularly lonely when the patient did not understand the solitary and fragile situation of the family caregiver:

He didn’t understand the amount of workload for me. I told the nurse: ‘can’t you do anything?’ She answered, ‘No, we can’t do anything as long as he doesn’t ask for it himself’. So, I felt they couldn’t help me, even though I desperately asked for it…. They don’t bother much about me…. (wife of patient, age 68).

Further, several of the family caregivers explained how living in a frangible life-situation close to an ill family member felt like they were sharing their suffering. Experiencing the suffering as a mutual perception was described both as a physical and an existential suffering. With regard to physical suffering, a few family caregivers explained how they felt bodily pain when the patient went through painful treatments. Moreover, sleepless nights were described as physically distressing when having the responsibility of nightly treatments or when family caregivers were lying awake listening to the patients’ breath. With regard to the existential suffering, it was described as living together in a constant state of emotional distress. Some family caregivers described it as having a dual suffering, both from the expected loss they were about to experience and from the current existential pain they experienced when their loved one was suffering:

It hurts … every time. When they are not taking her seriously... They must not do that to my love … it hurts. You just feel it, right? These strong bonds. As if you get attacked yourself (husband of patient, age 67).

The results indicate how dignity is achieved through finding meaning in the family caregiver role within the living environment, thereby emphasizing the importance of being seen and heard. Despite this, they suffer in a space of loneliness, which challenges their experience of dignity.

## Discussion

Family caregivers found dignity in caring for a loved one at home, but this was sometimes challenged by the demands of a medicalized environment. Their individuality and specific needs required recognition from HCPs, beyond their role as caregivers. Lack of support, knowledge about assistance, and feelings of loneliness, both at home and within the healthcare system, further complicated their emotional distress. This discussion will be structured around the three different dimensions of dignity – highlighting how family caregivers experience dignity and loss of dignity when taking care of a family member who is nearing end-of-life at home. Each dimension will be explored through a dialogue that interweaves our empirical findings with existing research. Additionally, each part will be discussed through the theoretical lens of Katie Eriksson’s concept of the *three living spaces*, seen in relation to the three circles within Figure 1.

The theme of the outer circle, *having a meaningful existence within the living environment*, served as a crucial source of preserving dignity. For the family caregivers, it was meaningful to follow the patients’ lead and to provide support in homely surroundings. This was associated with their relationship and how family caregivers felt both a great responsibility for the care given and gratitude to be able to provide such care to them. This rewarding aspect of the relationship is also discussed in Haan et al.,^17^ describing how caring for a close family member at home reveals a call to care due to their strong familial bonds. It appears that such unconditional and wholehearted relationships are crucial and meaningful yet also guided by family caregivers norms and social expectations. Similarly, Stenberg et al.^16^ highlight how family caregivers experience new insight into the caring relationship and establish a greater closeness when caring for a sick beloved at home. However, they also present how such closeness can create dilemmas for the family caregiver, balancing between the known and unknown in the situation and between demands and needs in their roles.^16^ This reflects our results – portraying how family caregivers were calmer and showing their feelings more openly within their own home rather than being a relative in an institution. However, it also reveals how the family caregivers were struggling to create meaningfulness and find sources of dignity in their home even when being exhausted and feeling vulnerable. This can be compared to a review undertaken by Woodman et al.,^18^ who emphasize how the meaning of home might change when caring for relatives at home and how it can be even turned into an impersonal and medicalized environment. Therefore, it is important for HCPs to meet vulnerable homeowners with a willingness to understand their individual rhythms of daily life in order to increase their feeling of security.^40^ Moreover, the living environment is associated with an emotional safety and an experience of dignity in the place you reside, described by the finish theorist Katie Eriksson.^41^ Her theoretical concept of *living spaces* of the human being constitutes three different living spaces that must be optimized while searching for health promotion. In the first one, the *physical living space,* Eriksson^41^ emphasizes that the primary function in life is to be ensconced in a concrete place to stay where you feel safe in a caring environment. Based on our findings, family caregivers experience dignity and a meaningful existence when they are independent and in charge of one’s physical environment. However, this was, in many cases, related to the mutual preferences of both the patient and the family caregiver. The meaningful existence and feeling of home were experienced when this was a shared experience with their ill family member. Therefore, it is important for HCPs to provide tailored support in the physical living space of *both* the patient and their family caregiver.

*Being seen and valued as a* family caregiver *in relation with others* was presented as the second theme within the middle circle and was found to be a crucial source of dignity when living in homely surroundings. This relational aspect holds different dimensions for the family caregivers, being both the important relationship with the patients as well as the relationship with HCP. First, within the patient – family caregiver relationship, the family caregiver experienced a dual wish to be both an independent family member in their everyday lives as well as a close caregiver. This reveals an ambiguity on the part of the family caregiver to choose between an ethical obligation towards the patient and a strong need to withhold their self-care. This imbalanced position is also described in previous research, indicating the difficult and ‘invisible’ life situation of family caregivers – alternating between the role as a family member and the role as a caregiver.^15^ Such a demanding task mirrors our findings, highlighting the family caregivers’ feeling of powerless and how they wished to spare and protect the patient. Therefore, the relationship with HCPs was of utmost importance for the family caregivers when caring for a beloved one in their home. These findings relate to recommendations by Danielsen et al.,^42^ who emphasized the important and necessary role of HCPs in enabling family caregivers to connect with the meaningfulness of caregiving. They indicated that HCPs must have a close and collaborative relationship with family caregivers in order to help them create moments of intimacy and significance with their ill family member. However, caring for a family member at home is also considered a complex matter and might create feelings of inadequacy for the family caregiver.^43^ This is in accordance with our study, yet surprisingly, we also found that family caregiver reject support from HCPs, despite their expressed wishes and needs to be seen and valued. We believe this paradoxical result is both related to a lack of knowledge regarding their opportunities for support as well as a promise made to their ill family member to reduce interference from HCPs. Barlund et al.^44^ add to this speculation by stating that family caregivers often reject professional support even though this is found to be decisive for them to feel secure in a home caring situation. As such, the loyalty towards the patient appears to be stronger for the family caregivers than the need to share worries and thoughts with the HCPs themselves. We believe this essential perspective is partially based on whether HCPs show a willingness and an effort to become familiar with the family caregiver, thereby valuing them as individuals. This matter can also be discussed as part of the second living space, the *psychosocial living space* by Eriksson.^41^ In this living space, interaction within relationships is of concern. In line with our study, indicating the importance of being seen and valued as a family caregiver, Eriksson emphasizes the power of a caring relationship where the human being feels validated and recognized in this living space. Our findings highlight that after practical help from HCPs, emotional support was vital. As such, the compassionate and gentle attitude of HCPs optimized the psychosocial living space and increased the sense of dignity for the family caregivers. The third theme within the centre of the circle, highlight how family caregivers were *suffering in a space of loneliness* and experienced a loss of dignity in homely surroundings when caring for a family member. The family caregivers expressed how they felt lonely due to the physical and existential burden they had to bear, as well as how they felt lonely within the healthcare system. This was related to the absence of a caring attitude by HCPs in several situations. According to Eriksson,^45^ this absence of cautiousness and understanding is associated with suffering for the family caregivers. Suffering has often been reduced to the language of physical suffering and is known as quite mute. This matches our empirical data and reveals that the voices of family caregivers are silenced and go unheard; occasionally, these voices are also difficult to hear in situations where family caregivers experience existential loneliness. Moreover, the family caregivers described how they share the existential suffering of the patient, defined as mutual emotional distress. This was related to the expected loss of a family member that provoked a lonely grieving process. However, it was also related to the current existential pain they experienced when witnessing the suffering of their loved ones. In the same manner, Beaver and Witham^46^ hold the view in their definition of ‘informal carer’, referring to someone who actively participates in sharing the patients’ illness experience at a practical and/or emotional level. This explanation also relates to how family caregivers and the patient experience a mutual influence concerning the illness and how the dignity of the patient and the family caregiver is strongly related. De Voogd et al.^28^ indicate how the dignity of family caregivers becomes crucial alongside the dignity of the patient. In their study, they found that when HCPs undermine the dignity of the patient, the family caregivers felt like their dignity was affected as well. In this situation, we might speak of ‘hidden patients’, which implies that family caregivers become additional victims of the patients’ disease.^22^ As such, it is crucial that HCPs are aware of the third living space, namely, the *existential living space*^41^ in order to recognize the hidden suffering of the family caregiver. In this living space, a meaningful and safe existence is of importance, a place where the human being can live harmoniously in accordance with his/her values, hopes, and needs.^41^ According to our findings, this harmonious living space is not optimal for the family caregivers, as they are barely offered support by HCPs. Furthermore, the limited support they received was experienced as superficial. Therefore, considering all three living spaces, we believe it is vital for family caregivers to meet HCPs who are genuinely willing and interested in upholding these living spaces for the family caregivers in order for them to reside in a space where they can experience dignity and meaningfulness.

## Methodological considerations

We found secondary analysis to be a suitable method when combining data from two primary studies in this article. A crucial reason for choosing this method is the possibility to analyze data at a later date, emphasizing that important data are not sufficiently focused on in the primary analysis.^47^ In particular when it comes to data of vulnerable populations or studies about sensitive topics, it is recommended to re-analyze data prior to engaging new participants.^48^ In this way, secondary analysis becomes a cumulative rather than a repetitive process.^33,49^ In both primary studies, bereaved family caregivers and current family caregivers of a family member nearing end-of-life were considered vulnerable. Crucial data considering family caregivers own experiences, were not analyzed, or used within the primary studies, and therefore considered important to reuse in this secondary analysis. Also, it is recommended to involve multiple researchers in a secondary analysis process, as the significance of data does not reside within the data itself, but rather is discovered by those seeking to find meaning within the material that is perceived to be data.^50^ However, some methodological limitations are worth noting.

A critical point could be that new researchers are not familiar with the context and authenticity of the data. Also, data may be interpreted differently by the same researcher with the passage of time as it influences the relationship of the primary researchers to the data.^51^ As a result, we cannot foresee how the informants would have shared their experiences with other researchers, and how the interviews were affected by the researcher. In addition, there is a difference between whether the informants were bereaved (study 1) or current family caregivers (study 2). Therefore, the data might have been affected by contextual variations and experiences that are processed differently related to the time when family caregivers were caring for a relative.

## Conclusions and implications

Family caregivers found dignity in their meaningful existence, derived from caring for a family member nearing end-of-life at home, given this was a shared preference. However, the demands of providing care in a medicalized, unsafe environment challenged this sense of dignity. Relational and psychosocial sources of dignity were vital, with the need for HCPs to view family caregivers as unique individuals, not just caregivers, and respond to their specific needs. The family caregivers’ lack of support and knowledge about available assistance, compounded by their loyalty to patients, could lead to emotional distress. Feeling lonely, both at home and within the healthcare system due to insufficient support, recognition, and the burden of care responsibilities, further exacerbated this distress. Our study adds to the base of knowledge concerning family caregivers’ experiences, sharing how they live in a frangible life-situation when caring for a family member nearing end-of-life at home in which high expectation and silenced voices are the reality. Based on these findings, we strongly suggest a practical value for emphasizing HCPs ethical training to reflect on attitudes and behaviours towards encouraging dignity-preserving care both in practice and education. Regarding policymaking, the acknowledgement of the family caregiver should be stronger defined within policy guidelines and documents. As this study does not fully explore the relationship between HCPs and family caregivers and its expectation in roles, we recommend further research on this topic.
